# Impact of Particulate Matter Exposure and Aerobic Exercise on Circulating Biomarkers of Oxidative Stress, Antioxidant Status, and Inflammation in Young and Aged Mice

**DOI:** 10.3390/life13101952

**Published:** 2023-09-23

**Authors:** Su-Youn Cho, Hee-Tae Roh

**Affiliations:** 1 Exercise Physiology Laboratory, Department of Physical Education, Yonsei University, Seoul 03722, Republic of Korea; 2 Division of Sports Science, College of Arts and Sports, Sun Moon University, 70 Sunmoon-ro 221 beon-gil, Tangjeong-myeon, Asan-si 31460, Republic of Korea

**Keywords:** particulate matter, exercise training, oxidative stress, inflammation, aging

## Abstract

Exposure to particulate matter (PM) and exercise training can have antagonistic effects on inflammatory responses and the balance between pro-oxidants and antioxidants in the body. However, the underlying mechanisms of these effects remain unclear. This study aimed to investigate the effects of PM exposure and aerobic exercise training on oxidative stress, antioxidant status, and inflammation in mice of different ages. Two groups of male C57BL/6 mice, comprising forty 1-month-old and forty 12-month-old mice, were exposed to either PM or exercise training or both for 8 weeks. PM exposure led to significantly higher 8-hydroxydeoxyguanosine (8-OHdG), malondialdehyde (MDA), interleukin-1β (IL-1β), interleukin-6 (IL-6), and tumor necrosis factor α (TNF-α) levels (*p* < 0.05) and significantly lower superoxide dismutase (SOD) and catalase (CAT) activities (*p* < 0.05) in both age groups exposed to PM compared to the control groups. Conversely, aerobic exercise training led to significantly lower 8-OHdG, MDA, IL-1β, IL-6, and TNF-α levels (*p* < 0.05) and significantly higher SOD and CAT activities (*p* < 0.05) in both age groups receiving exercise training, compared to those exposed to PM. Moreover, young mice in the exercise training and PM group showed significantly lower 8-OHdG, MDA, and IL-1β levels (*p* < 0.05) and significantly higher SOD and CAT activities (*p* < 0.05) than young mice in the PM exposure group. However, these levels did not vary significantly between the group of old mice that either received exercise training or exposure to PM. Our results suggest that while PM exposure could cause pro-oxidant/antioxidant imbalances and inflammatory responses, regular aerobic exercise could ameliorate these negative effects, although these vary with age. Nevertheless, the antioxidant and anti-inflammatory effects of exercise were countered by PM exposure, especially in older mice.

## 1. Introduction

At present, particulate matter (PM) exposure is considered the most important environmental factor affecting public health, contributing significantly to global mortality and disease [1]. PM refers to extremely small particles that float in the air or are blown by the wind in the environment; these may include chemical compounds, such as nitrates, sulfates, and polycyclic aromatic hydrocarbons, which induce cytotoxicity and destroy or damage cells in the body [2]. Long-term exposure to PM could cause respiratory diseases, ranging from asthma to chronic obstructive pulmonary disease and lung cancer, metabolic diseases, cardiovascular diseases, including hypertension, myocardial infarction, coronary artery disease, and diabetes, and various other diseases in different organs of the body, including the brain, skin, pancreas, and uterus [3,4,5]. The pathogenic mechanism of PM is attributed to the strong inflammatory responses and reduced immunity caused by oxidative stress (OS). The inhalation of PM in the respiratory tract induces OS, which subsequently triggers an inflammatory response, activating the immune system [6]. The consequent overexpression of inflammatory cytokines via cellular signaling causes DNA damage in various tissues, leading to apoptosis; thus, PM ultimately leads to a multitude of diseases [7].

The health risk posed by PM is closely associated with age. Individuals older than 65 years are more susceptible to a higher prevalence of cardiovascular and respiratory diseases caused by exposure to PM [8]. In addition, long-term PM exposure greatly increases the rate of early mortality in younger adults due to various diseases, including cancer [9,10]. In older individuals, high PM levels pose an increased health risk individuals because of the reduced function of their oxidant-antioxidant system [11,12]. The human body has an antioxidant system that comprises various antioxidant enzymes, including catalase (CAT), glutathione peroxidase (GPX), and superoxide dismutase (SOD), which contribute to the maintenance of homeostasis by removing free radicals and reactive oxygen species (ROS) from the matrix of the mitochondria in certain tissues [13,14]. With age, the activity of the antioxidant enzymes decreases, while free radicals and ROS are excessively produced in the body [15], disrupting the balance of the antioxidant mechanism and thus enabling a more pronounced generation of PM-induced OS. Moreover, in an aging body, the immune system weakens, leading to a diminished ability to effectively combat various antigens. Simultaneously, the production of various inflammatory markers increases [16,17], leading to deterioration of the physical functions of the body and an increased risk of the development of geriatric diseases, including cardiovascular diseases [18]. Thus, PM exposure in older adults with a concurrent increase in OS and a decline in immunity could accelerate the aging process, ultimately increasing the risk of mortality.

Regular exercise improves the oxidant-antioxidant imbalance and exerts anti-inflammatory effects, preventing the onset of pathological symptoms, including OS and systemic inflammation [19,20]. Regular exercise can activate nuclear factor kappa-B (NF-κB) pathways and increase the resistance to cellular stress by activating mitogen-activated protein kinases, which in turn increase the activity of various antioxidant enzymes, such as SOD, CAT, GPX, and glutathione reductase (GR). These enzymes reduce the levels of systemic inflammatory factors, such as C-reactive protein, interleukin-6 (IL-6), and tumor necrosis factor α (TNF-α), and promote the expression of anti-inflammatory cytokines [21,22]. Recently, several studies have shown that exercise could reduce the negative effects of PM exposure and the risk of related diseases [23,24]. So et al. reported that short-term PM exposure induced OS, pro-inflammatory responses, and apoptosis in the lungs; however, regular exercise alleviated the PM-induced OS and the pro-inflammatory responses [25]. Nevertheless, while researchers have suggested that the exercise-induced oxidant-antioxidant balance and inflammatory responses may vary according to the age of individuals [26,27], only a limited number of studies have investigated the responses of individuals of different ages to the antagonistic effects of PM exposure and exercise. Therefore, in the present study, we aimed to determine the effects of PM exposure and aerobic exercise training on the OS, antioxidant status, and inflammatory responses in mice of different ages.

## 2. Materials and Methods

### 2.1. Experimental Animals and Maintenance

Forty 1-month-old (Young, *n* = 40) and forty 12-month-old (Aged, *n* = 40) male C57BL/6 mice were purchased from Samtako Bio-Korea Inc. (Gyeonggi-do, Osan, Republic of Korea). The mice were housed in plastic cages with each cage accommodating four mice, and were maintained in an animal lab with a constant supply of sterile air using a high-efficiency particulate arrestant filter, and in positive pressure conditions to block the entry of contaminated external air. The mice were bred at a temperature of 22 °C ± 2 °C, which was automatically controlled, a relative humidity of 55% ± 5%, and under 12/12 h light/dark cycles (07:00 lights on; 19:00 lights off) and had ad libitum access to food (69.41% carbohydrate, 6.52% fat, and 24.34% protein, Research Diets, New Brunswick, NJ, USA) and drinking water. After a week of acclimatization, the mice were grouped into the young control (YCO, *n* = 10), aged control (ACO, *n* = 10), young PM administration (YPM, *n* = 10), aged PM administration (APM, *n* = 10), young exercise (YEX, *n* = 10), aged exercise (AEX, *n* = 10), young PM administration and exercise (YPMEX, *n* = 10), and aged PM administration and exercise groups (APMEX, *n* = 10). The animal experiments were approved by the National Research Foundation of Korea (2020S1A5A8047345).

### 2.2. PM Intervention

PM was administered to the YPM, APM, YPMEX, and APMEX groups three times a week for 8 weeks. The PM used in the experiment was Fine dust (PM_10_-like, ERM-CZ120), which was purchased from Sigma-Aldrich (St. Louis, MO, USA), and whose PM_10_-like composition and concentration are certified by the European Reference Materials (ERM). Following the methodology described by Bai and van Eeden [28], 15 μg of PM was suspended in 200 μL of saline and 0.5 μg of PM per weight (in g) of mice was injected into the relevant groups via the tail vein. For the YCO, ACO, YEX, and AEX groups, 200 μL of saline was intravenously administered via the tail vein.

### 2.3. Aerobic Exercise Intervention

Mice in the YEX, AEX, YPMEX, and APMEX groups ran for 40 min on an animal treadmill 5 times a week for 8 weeks, without being electrically stimulated. All mice that were randomly assigned to the exercise groups ran on a treadmill with a 0% slope for 10 min for 3 days at a speed varying between 5 to 10 m/min for adaptation. The warm-up and cool-down consisted of running at 5 m/min for 5 min. The main exercise consisted of running at 8 m/min for 30 min from week 1 to 4, followed by an increase in exercise intensity, running for 20 min at 10 m/min from week 5 to 8.

### 2.4. Blood Sampling and Analysis

For blood sampling, mice were anesthetized using ethyl ether, and 5 mL of blood was collected from the abdominal inferior vena cava. The collected blood was centrifuged (3000 rpm at 4 °C) to isolate the serum and stored at –80 °C for subsequent analysis. The serum 8-hydroxydeoxyguanosine (8-OHdG; #ab201734; Abcam, Cambridge, UK), malondialdehyde (MDA; #MBS741034; MyBioSource, San Diego, CA, USA), SOD (#MBS034842, MyBioSource), CAT (#MBS704962, MyBioSource), interleukin-1β (IL-1β; #DY401, R&D Systems, Minneapolis, MN, USA), IL-6 (#DY406, R&D Systems), and TNF-α (#DY410, R&D Systems) levels were measured using enzyme-linked immunosorbent assays by following the manufacturers’ instructions.

### 2.5. Statistical Analysis

Statistical analysis was performed using SPSS version 29.0 for Windows (IBM Corp., Armonk, NY, USA). Data are expressed as the mean ± standard deviation (SD) for all dependent variables. Various OS, antioxidant status, and inflammatory biomarkers were compared between age groups and intervention conditions using a two-way analysis of variance (ANOVA). The factors considered were age (young or old) and intervention condition (control, PM exposure, exercise, and PM exposure and exercise). When significant interactions occurred, the main effects were assessed using one-way ANOVA. Pearson’s correlation analysis was performed to assess the relationship between OS, antioxidant status, and inflammatory biomarkers. Multiple linear regression analysis was performed to analyze the association between OS, antioxidant status, and inflammation with age, PM exposure, and exercise. Statistical significance was set at *p* < 0.05.

## 3. Results

### 3.1. Changes in Biomarkers of Oxidative Stress

#### 3.1.1. Changes in Serum 8-OHdG Levels

**Figure 1:**
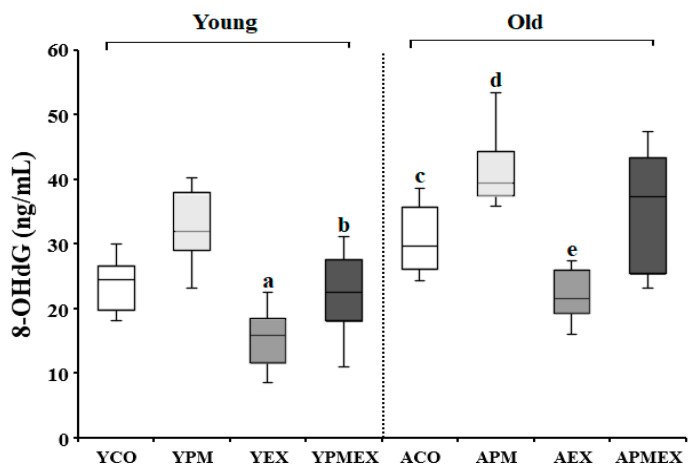
Changes in serum 8-OHdG levels with PM exposure and aerobic exercise. Data are expressed as mean ± SD. YCO: young control group, ACO: aged control group, YPM: young PM administration group, APM: aged PM administration group, YEX: young exercise group, AEX: aged exercise group, YPMEX: young PM administration and exercise group, APMEX: aged PM administration and exercise group, ^a^ versus YCO (*p* < 0.05), ^b^ versus ACO (*p* < 0.05), ^c^ versus YCO, YPM, and AEX (*p* < 0.05), ^d^ versus ACO and APM (*p* < 0.05), ^e^ versus YPM and APMEX (*p* < 0.05).

Changes in serum 8-OHdG levels with PM exposure and aerobic exercise are shown in Figure 1. Two-way ANOVA showed significant interaction effects in serum 8-OHdG levels (*F* = 22.147, *p* < 0.001). Post hoc analysis revealed that the YPM and APM groups showed significantly higher 8-OHdG levels than the YCO and ACO groups, respectively (*p* < 0.05). In contrast, the YEX and AEX groups that performed aerobic exercise showed significantly lower 8-OHdG levels compared to the YCO and ACO groups as well as the YPM and APM groups, respectively (*p* < 0.05). While the YPMEX group showed significantly lower 8-OHdG levels than the YPM group (*p* < 0.05), the APMEX group showed no significant difference compared to the APM group. Moreover, the YEX and YPMEX groups showed significantly lower 8-OHdG levels than the AEX and APMEX groups, respectively (*p* < 0.05).

#### 3.1.2. Changes in Serum MDA Levels

**Figure 2:**
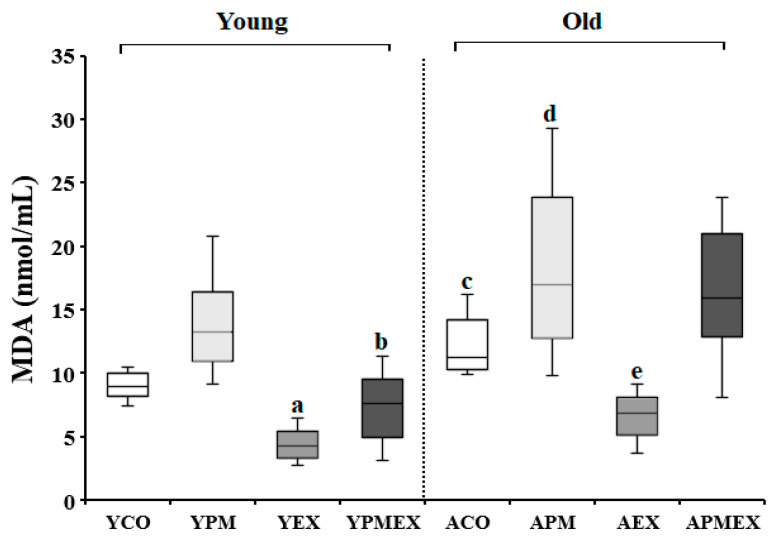
Changes in serum MDA levels with PM exposure and aerobic exercise. Data are expressed as mean ± SD. YCO: young control group, ACO: aged control group, YPM: young PM administration group, APM: aged PM administration group, YEX: young exercise group, AEX: aged exercise group, YPMEX: young PM administration and exercise group, APMEX: aged PM administration and exercise group, ^a^ versus YCO (*p* < 0.05), ^b^ versus ACO (*p* < 0.05), ^c^ versus YCO and YPM (*p* < 0.05), ^d^ versus ACO and APM (*p* < 0.05), ^e^ versus YPM and APMEX (*p* < 0.05).

Changes in serum MDA levels with PM exposure and aerobic exercise are shown in Figure 2. Two-way ANOVA showed significant interaction effects in serum MDA levels (*F* = 17.386, *p* < 0.001). Post hoc analysis revealed that the YPM and APM groups showed significantly higher MDA levels than the YCO and ACO groups, respectively (*p* < 0.05). Conversely, the YEX and AEX groups that performed aerobic exercise showed significantly lower MDA levels than those in the YCO and ACO groups as well as the YPM and APM groups, respectively (*p* < 0.05). The YPMEX group showed significantly lower MDA levels than the YPM group (*p* < 0.05), whereas no significant differences were observed between the APMEX and APM groups. Furthermore, the YPMEX group showed significantly lower MDA levels than the APMEX group (*p* < 0.05).

### 3.2. Changes in Biomarkers of Antioxidant Status

#### 3.2.1. Changes in Serum SOD Activities

**Figure 3:**
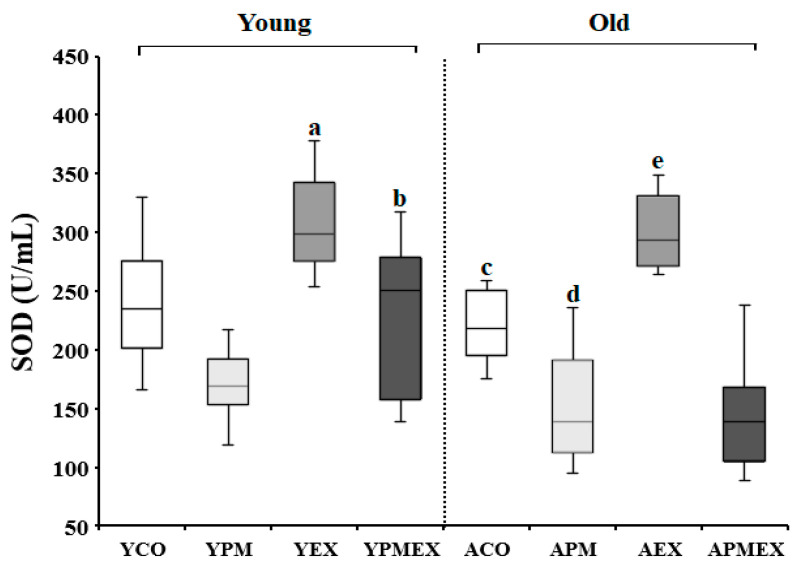
Changes in serum SOD activities with PM exposure and aerobic exercise. Data are expressed as mean ± SD. YCO: young control group, ACO: aged control group, YPM: young PM administration group, APM: aged PM administration group, YEX: young exercise group, AEX: aged exercise group, YPMEX: young PM administration and exercise group, APMEX: aged PM administration and exercise group, ^a^ versus YCO (*p* < 0.05), ^b^ versus ACO (*p* < 0.05), ^c^ versus YCO and YPM (*p* < 0.05), ^d^ versus ACO and APM (*p* < 0.05), ^e^ versus YPM and APMEX (*p* < 0.05).

Changes in serum SOD activities with PM exposure and aerobic exercise are shown in Figure 3. Two-way ANOVA showed significant interaction effects in serum SOD activities (*F* = 19.380, *p* < 0.001). Post hoc analysis results revealed that the YPM and APM groups showed significantly lower SOD activities than the YCO and ACO groups, respectively (*p* < 0.05). However, the YEX and AEX groups that performed aerobic exercise showed significantly higher SOD activities than the YCO and ACO groups, as well as the YPM and APM groups, respectively (*p* < 0.05). Moreover, the YPMEX group showed significantly higher SOD activities than the YPM group (*p* < 0.05), whereas no significant difference was observed between the APMEX and APM groups. The YPMEX group showed significantly higher SOD activities than the APMEX group (*p* < 0.05).

#### 3.2.2. Changes in Serum CAT Activity

**Figure 4:**
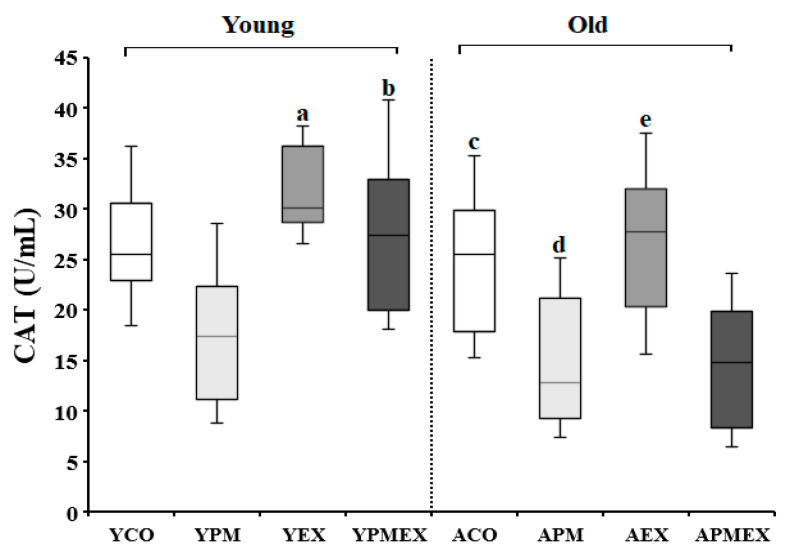
Changes in serum CAT activities with PM exposure and aerobic exercise. Data are expressed as mean ± SD. YCO: young control group, ACO: aged control group, YPM: young PM administration group, APM: aged PM administration group, YEX: young exercise group, AEX: aged exercise group, YPMEX: young PM administration and exercise group, APMEX: aged PM administration and exercise group, ^a^ versus YCO (*p* < 0.05), ^b^ versus ACO (*p* < 0.05), ^c^ versus YPM (*p* < 0.05), ^d^ versus APM (*p* < 0.05), ^e^ versus YPM and APMEX (*p* < 0.05).

Changes in serum CAT activities with PM exposure and aerobic exercise are shown in Figure 4. Two-way ANOVA showed significant interaction effects in serum CAT activities (*F* = 10.841, *p* < 0.001). Post hoc analysis test revealed that the YPM and APM groups showed significantly lower CAT activities than the YCO and ACO groups, respectively (*p* < 0.05). In contrast, the YEX and AEX groups that performed aerobic exercise showed significantly higher CAT activities than the YPM and APM groups, respectively (*p* < 0.05). The YPMEX group showed significantly higher CAT activities than the YPM group (*p* < 0.05), where no significant differences were observed between the APMEX and APM groups. Additionally, the YPMEX group showed significantly higher CAT activities than the APMEX group (*p* < 0.05).

### 3.3. Changes in Biomarkers of Inflammation

#### 3.3.1. Changes in Serum IL-1β Levels

**Figure 5:**
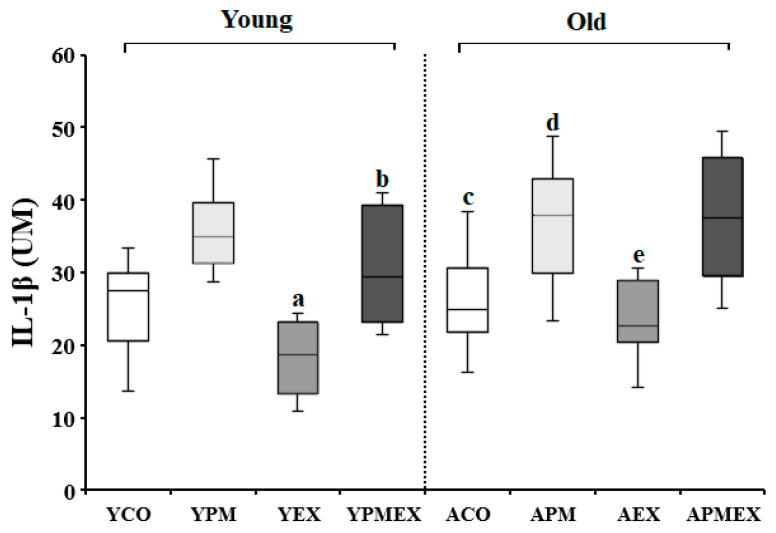
Changes in serum IL-1β levels with PM exposure and aerobic exercise. Data are expressed as mean ± SD. YCO: young control group, ACO: aged control group, YPM: young PM administration group, APM: aged PM administration group, YEX: young exercise group, AEX: aged exercise group, YPMEX: young PM administration and exercise group, APMEX: aged PM administration and exercise group, ^a^ versus YCO (*p* < 0.05), ^b^ versus ACO (*p* < 0.05), ^c^ versus YPM (*p* < 0.05), ^d^ versus APM (*p* < 0.05), ^e^ versus YPM and APMEX (*p* < 0.05).

Changes in serum IL-1β levels with PM exposure and aerobic exercise are shown in Figure 5. Two-way ANOVA showed significant interaction effects in serum IL-1β levels (*F* = 11.287, *p* < 0.001). Post hoc analysis revealed that the YPM and APM groups showed significantly higher IL-1β levels than the YCO and ACO groups, respectively (*p* < 0.05). In contrast, the YEX and AEX groups that performed aerobic exercise showed significantly lower IL-1β levels than the YPM and APM groups, respectively (*p* < 0.05). The YPMEX group showed significantly lower IL-1β levels than the YPM group (*p* < 0.05), whereas no significant differences were observed between the APMEX and APM groups. Further, the YPMEX group showed significantly lower IL-1β levels than the APMEX group (*p* < 0.05).

#### 3.3.2. Changes in Serum IL-6 Levels

**Figure 6:**
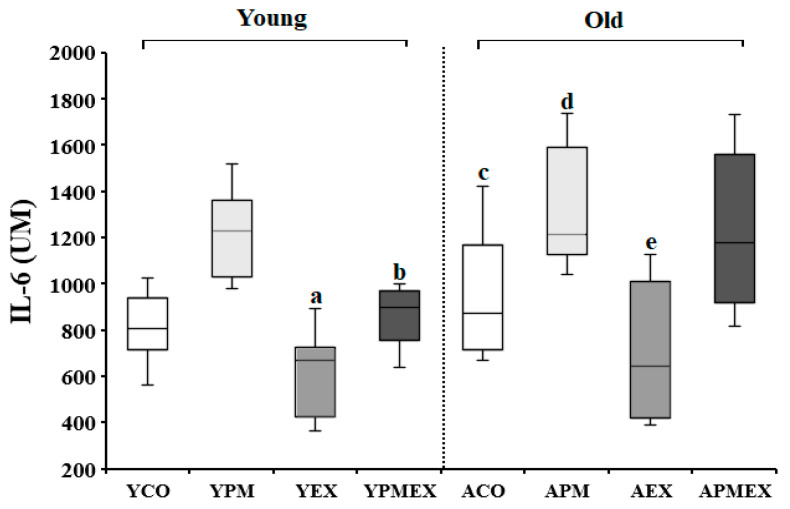
Changes in serum IL-6 levels with PM exposure and aerobic exercise. Data are expressed as mean ± SD. YCO: young control group, ACO: aged control group, YPM: young PM administration group, APM: aged PM administration group, YEX: young exercise group, AEX: aged exercise group, YPMEX: young PM administration and exercise group, APMEX: aged PM administration and exercise group, ^a^ versus YCO (*p* < 0.05), ^b^ versus ACO (*p* < 0.05), ^c^ versus YPM (*p* < 0.05), ^d^ versus APM (*p* < 0.05), ^e^ versus APMEX (*p* < 0.05).

Changes in serum IL-6 levels with PM exposure and aerobic exercise are shown in Figure 6. Two-way ANOVA showed significant interaction effects in serum IL-6 levels (*F* = 11.813, *p* < 0.001). Post hoc analysis revealed that the YPM and APM groups showed significantly higher IL-6 levels than the YCO and ACO groups, respectively (*p* < 0.05). Conversely, the YEX and AEX groups that performed aerobic exercise showed significantly lower IL-6 levels than the YPM and APM groups, respectively (*p* < 0.05). However, the YPMEX and APMEX groups did not show significant differences compared to the YPM and APM groups, respectively. Moreover, the YPMEX group showed significantly lower IL-6 levels than the APMEX group (*p* < 0.05).

#### 3.3.3. Changes in Serum TNF-α Levels

**Figure 7:**
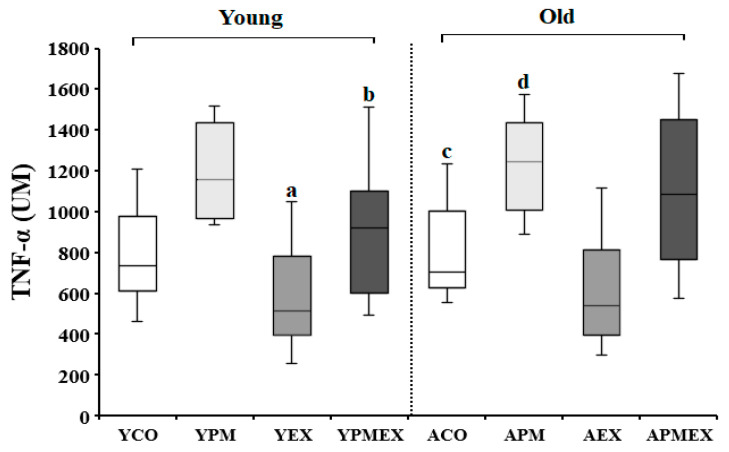
Changes in serum TNF-α levels with PM exposure and aerobic exercise. Data are expressed as mean ± SD. YCO: young control group, ACO: aged control group, YPM: young PM administration group, APM: aged PM administration group, YEX: young exercise group, AEX: aged exercise group, YPMEX: young PM administration and exercise group, APMEX: aged PM administration and exercise group, ^a^ versus YCO (*p* < 0.05), ^b^ versus ACO (*p* < 0.05), ^c^ versus YPM (*p* < 0.05), ^d^ versus APM (*p* < 0.05).

Changes in serum TNF-α levels with PM exposure and aerobic exercise are shown in Figure 7. Two-way ANOVA showed significant interaction effects in serum TNF-α levels (*F* = 8.704, *p* < 0.001). Post hoc analysis revealed that the YPM and APM groups showed significantly higher TNF-α levels than the YCO and ACO groups, respectively (*p* < 0.05). Conversely, the YEX and AEX groups that performed aerobic exercise showed significantly lower TNF-α levels than the YPM and APM groups, respectively (*p* < 0.05). Moreover, the APMEX group showed significantly higher TNF-α levels than the AEX group (*p* < 0.05), whereas the YPMEX group showed no significant difference compared to the YEX group.

### 3.4. Correlation between OS, Antioxidant Status, and Inflammation Biomarkers

The results of the correlation analysis using Pearson’s correlation coefficient to investigate the correlation and direction of each measurement variable are shown in Table 1. There was a significant positive correlation between OS (serum 8-OHdG and MDA levels) and inflammatory (serum IL-1β, IL-6, and TNF-α levels) biomarkers (*p* < 0.05). The antioxidant status (serum SOD and CAT activities) biomarkers showed a significant negative correlation between OS and inflammatory biomarkers (*p* < 0.05).

### 3.5. Multiple Linear Regression Analysis of Dependent Variables

The results of the multiple linear regression analysis conducted to investigate the effects of age, PM exposure, and exercise on dependent variables are shown in Table 2. In terms of serum 8-OHdG and MDA levels, which are OS biomarkers, age (*p* < 0.001 and *p* < 0.001, respectively) and PM (*p* < 0.001 and *p* < 0.001, respectively) exhibited a positive effect, whereas exercise (*p* < 0.001 and *p* < 0.001, respectively) had a negative effect. For serum SOD and CAT activities, which are biomarkers of antioxidant status, age (*p* = 0.002 and *p* < 0.001, respectively) and PM (*p* < 0.001 and *p* < 0.001, respectively) exhibited negative effects, whereas exercise (*p* < 0.001 and *p* = 0.004, respectively) had positive effects. For serum IL-1β and IL-6 levels, which are inflammatory biomarkers, age (*p* = 0.018 and *p* = 0.005, respectively) and PM (*p* < 0.001 and *p* < 0.001, respectively) had a positive effect, whereas exercise (*p* = 0.019 and *p* < 0.001, respectively) had a negative effect. For serum TNF-α levels, PM (*p* < 0.001) appeared to have a positive effect, whereas exercise (*p* = 0.001) had a negative effect.

## 4. Discussion

Various epidemiological studies have demonstrated the correlation between PM exposure and morbidity and mortality related to respiratory and cardiovascular diseases [29,30]. The exact mechanism via which PM deteriorates health remains unclear; however, most studies suggest that the association between the generation of ROS and the consequent OS-induced plays a central role in generating PM-induced cytotoxicity [31]. PM comprises harmful substances, such as polycyclic aromatic hydrocarbons and heavy metals, which generate ROS via the redox cycle [32]. Once generated, the ROS reacts with highly unsaturated fatty acids in the cellular membranes to facilitate the production of lipid peroxides, which result in OS and various diseases, including cancer and cardiovascular diseases [6,11,32]. In this study, the serum levels of 8-OHdG and MDA were measured to verify the effects of PM exposure and aerobic exercise on OS in mice of different ages. 8-OHdG is the main product of the oxidative damage caused by free radicals [33]; it is not only used as a biomarker of OS but also as an indicator of endogenous oxidative DNA damage and the risk of various diseases, including cancer [34]. MDA is a marker of lipid peroxidation that induces cytotoxicity and cellular stress. It is produced when polyunsaturated fatty acids are damaged by ROS, and its use as an indicator of in vitro and in vivo OS generation has been suggested [35,36]. In the present study, our results revealed that the levels of 8-OHdG and MDA were significantly higher in the YPM and APM group mice than in the YCO and ACO group mice, respectively. In a cohort study conducted on 97 older adults, a positive correlation was identified between PM exposure and MDA levels in the participants [11]. PM exposure has also been shown to increase the 8-OHdG levels in mice [37] and in an in vitro culture of bronchial epithelial cells [38]. In our study, a multiple linear regression analysis revealed that PM exposure had a significant positive effect on serum 8-OHdG and MDA levels, which are OS biomarkers. This result suggests that exposure to PM promotes the induction of OS in the body, consistent with the results of previous studies [11,37,38] reporting that PM exposure may be the main cause of OS in the human body and animal models.

The generation of OS and inflammatory responses are positively correlated. Consistently, in this study, the levels of OS biomarkers and pro-inflammatory factors (IL-1β, IL-6, and TNF-α) were significantly higher in the YPM and APM mice than in the YCO and ACO mice, respectively. In addition, there was a significant positive correlation between OS and inflammatory biomarkers. Inflammation is generally part of the physical response to internal and external stimuli; however, a chronic state of inflammation can induce the pathogenesis of various diseases, including diabetes, autoimmune disease, and cancer, and can further accelerate disease progression. IL-1β is a pro-inflammatory cytokine that not only facilitates the inflammatory responses in the aorta and influences the proliferation of vascular smooth muscle cells, but also participates in vascular endothelial dysfunction and facilitates atherosclerosis [39]. IL-6 is a cytokine that affects various other cellular functions; it acts as a precursor of inflammation, induces inflammatory responses upon tumorigenesis and infection, increases the levels of growth factors, and triggers angiogenesis, ultimately inducing the abnormal proliferation of epithelial and extracellular matrix cells [40]. TNF-α is a representative inflammatory cytokine that mainly participates in the early inflammatory response [41]. An increase in blood TNF-α levels raises the risk of pulmonary disease and rheumatic arthritis, cardiac insufficiency, leukemia, and sepsis [42]. Sahu et al. reported that the brains of mice that suffered long-term exposure to PM had increased levels of cytokines, including TNF-α, IL-6, IL-1β, IFN-γ, and MIP-3α [43]. In addition, Zeng et al. reported that PM exposure increased ROS levels, activating the NF-κB pathway, which regulates the transcription of the genes encoding inflammatory cytokines, thus increasing the levels of IL-6, IL-8, and IL-1β [44].

An association between an increased OS generation and inflammatory responses as a result of PM exposure and a decreased antioxidant function has been suggested [6,45,46]. Under normal conditions, cells use various enzymatic and non-enzymatic defense mechanisms in response to free radicals, which constitute the key defense mechanisms against oxidative damage to cells and organs. However, PM exposure reduces the SOD and CAT activities both in vivo and in vitro [45,46]. In addition, Wei et al. reported that PM exposure significantly decreases the activity of antioxidant enzymes, such as SOD and CAT, and the total antioxidant capacity (T-AOC) in humans, indicating the role of PM in the suppression of the antioxidant system in the human body [47]. Consistently, in this study, the SOD and CAT activities were significantly lower in the YPM and APM groups than in the YCO and ACO groups, respectively, which may have affected OS and the inflammatory marker levels. In addition, our multiple linear regression analysis revealed that PM exposure exhibited a significantly negative effect on serum SOD and CAT activities, suggesting that PM exposure can induce a decrease in antioxidant enzyme activities.

Regular exercise exerts anti-inflammatory effects and enhances antioxidant abilities, thereby preventing certain events associated with pathologies, such as chronically high levels of OS and inflammatory responses [19]. In this study, the levels of IL-1β, IL-6, and TNF-α were significantly lower in the YEX and AEX groups than in the YPM and APM groups, respectively. Furthermore, 8-OHdG and MDA levels, along with the SOD activities in the YEX and AEX groups, were lower and higher, respectively, than those in the YPM and APM groups as well as the YCO and ACO groups, respectively. Moreover, our multiple linear regression analysis also demonstrated that exercise had a significantly negative effect on OS and inflammatory biomarkers, but had a significantly positive effect on antioxidant status biomarkers. These results indicate that, in addition to its antioxidant and anti-inflammatory effects, regular exercise may improve PM-related inflammatory responses and the pro-oxidant/antioxidant imbalance.

Exercise increases the transcription of genes encoding antioxidant enzymes, such as SOD, CAT, and glutathione peroxidase (GPX), thus helping improve the antioxidant system of the body [19,48]. Exercise can also enhance the circulation of anti-inflammatory cytokines and reduce the pro-inflammatory state related to disease [25,49]. Several previous studies have attempted to confirm whether the positive effects of exercise can be retained in high PM conditions. So et al. showed that PM exposure increased the levels of pro-inflammatory factors, such as IL-6 and TNF-α, whereas aerobic exercise reduced the expression of these factors. They also suggested that exercise could exert a protective effect against PM-induced inflammatory responses in the lungs [25]. Conversely, Marmett et al. reported that exercise could increase the levels of SOD and glutathione and decrease the levels of IL-1β and lipid peroxidation in cardiac tissues and skeletal muscles; however, PM exposure reduced these beneficial effects [24]. In the present study, SOD and CAT activities were significantly higher and the levels of 8-OHdG, MDA, and IL-1β were significantly lower in the YPMEX group than in the YPM group. The results complement those of previous studies reporting the role of exercise in improving the negative impact of PM on the oxidant-antioxidant balance and inflammatory responses.

Notably, in the present study, no significant differences in any measured variable were observed between the APMEX and the APM groups. Moreover, significantly lower SOD and CAT activities, but higher MDA, IL-1β, and IL-6 levels were identified in the APMEX group than in the YPMEX mice. Additionally, the multiple linear regression analysis revealed that, unlike exercise, age had a significantly positive effect on OS and inflammatory biomarkers, but a significantly negative effect on antioxidant status biomarkers. These results indicate that age could be a significant factor affecting the influence of PM exposure and exercise training on the pro-oxidant/antioxidant balance and inflammatory responses. Previous studies on humans and animal models have shown that aging can induce changes in the levels of transcription factors, such as NF-κB and activator protein 1, which plays a crucial role in antioxidant enzymatic activities that could reduce the adaptive effects of regular exercise on antioxidant enzymes. Bouzid et al. showed that the antioxidant enzymatic activities and the lipid peroxidation levels were similar in both sedentary young and old participants practicing exercise regularly [26], and emphasized the role of regular exercise in delaying aging-related damage to the body. They also reported that SOD and GR activities were significantly lower and MDA levels were significantly higher in old participants practicing regular exercise than in younger ones practicing regular exercise, in a resting state, respectively, which indicates that the beneficial effects of exercise could be reduced by the process of aging. The results obtained for the young mice in the present study also demonstrated that the negative impact of PM on the oxidant-antioxidant functions and inflammatory responses could be mitigated by regular aerobic exercise, whereas such effects were not observed in older mice. These results indicate the potential age-related variation in the beneficial effects of exercise under high PM conditions.

This study’s strength lies in that it investigated the effects of PM exposure and regular aerobic exercise on oxidant/antioxidant balance and inflammation using circulating peripheral markers across age groups. The medical significance of the results of this study suggests that the antioxidant and anti-inflammatory effects of exercise may appear in younger age groups even when exposed to PM, but these benefits may not be observed in older age groups. Verification of circulating peripheral markers is believed to have high utility in human studies and can contribute to the performance of many follow-up studies. This study has several limitations. First, the mice used in this study were all male; thus, the influence of sex on the results could not be evaluated. Previous studies have shown that resting antioxidant levels in females may be higher than those in males [50], and sex has been suggested as an important factor in changes in mitochondrial function in response to OS [51]. Future studies will need to consider sex-based differences. Second, the intensity of the exercise performed by each mouse could not be controlled. Considering that the regulation of oxidant/antioxidant status by exercise training is dependent on exercise intensity and may differ depending on the type of exercise, future studies will need to investigate various exercise intensities and types of exercise. Third, the effects of PM exposure and regular aerobic exercise on the OS, antioxidant status, and inflammation were assessed based on circulating peripheral markers. In future studies, molecular and proteomic analyses should be performed in specific organs.

## 5. Conclusions

In summary, our results showed that, while PM exposure can cause pro-oxidant/antioxidant imbalances and inflammatory responses regardless of age, regular aerobic exercise can ameliorate these negative effects. Nevertheless, the antioxidant and anti-inflammatory effects of exercise were reduced in PM exposure conditions, and this trend was the most pronounced in older mice. This result is indicative of potential age-related variations in the beneficial effects of exercise in PM exposure conditions.

## Figures and Tables

**Table 1:** Results of Pearson’s correlation analysis between each variable.

	8-OHdG	MDA	SOD	CAT	IL-1β	IL-6	TNF-α
8-OHdG	1						
MDA	0.565 *	1					
SOD	−0.611 *	−0.631 *	1				
CAT	−0.567 *	−0.590 *	0.607 *	1			
IL-1β	0.599 *	0.519 *	−0.561 *	−0.541 *	1		
IL-6	0.602 *	0.657 *	−0.571 *	−0.473 *	0.539 *	1	
TNF-α	0.490 *	0.507 *	−0.491 *	−0.471 *	0.510 *	0.505 *	1

8-OHdG: 8-hydroxydeoxyguanosine, MDA: malondialdehyde, SOD: superoxide dismutase, CAT: catalase, IL-1β: interleukin-1β, IL-6: interleukin-6, TNF-α: tumor necrosis factor α, * *p* < 0.05.

**Table 2:** Multiple linear regression analysis of dependent variables.

Dependent Variables	Explanatory Variables	B	Beta	t	*p*	*F*
8-OHdG	Age	8.763	0.458	6.902	<0.001	50.334 *
	PM	9.858	0.515	7.765	<0.001	
	Exercise	−8.333	−0.436	−6.563	<0.001	
	*R* = 0.816, *R*^2^ = 0.665, Adjusted *R*^2^ = 0.652, Durbin-Watson = 1.784					
MDA	Age	4.657	0.412	5.513	<0.001	34.291 *
	PM	5.516	0.488	6.530	<0.001	
	Exercise	−4.615	−0.408	−5.463	<0.001	
	*R* = 0.758, *R*^2^ = 0.575, Adjusted *R*^2^ = 0.558, Durbin-Watson = 1.567					
SOD	Age	−34.620	−0.243	−3.292	0.002	35.757 *
	PM	−91.670	−0.644	−8.716	<0.001	
	Exercise	47.580	0.334	4.524	<0.001	
	*R* = 0.765, *R*^2^ = 0.585, Adjusted *R*^2^ = 0.569, Durbin-Watson = 1.688					
CAT	Age	−5.513	−0.321	−3.761	<0.001	20.435 *
	PM	−9.072	−0.528	−6.189	<0.001	
	Exercise	4.363	0.254	2.97	0.004	
	*R* = 0.668, *R*^2^ = 0.446, Adjusted *R*^2^ = 0.425, Durbin-Watson = 1.673					
IL-1β	Age	3.603	0.197	2.409	0.018	24.744 *
	PM	11.838	0.646	7.915	<0.001	
	Exercise	−3.597	−0.196	−2.405	0.019	
	*R* = 0.703, *R*^2^ = 0.494, Adjusted *R*^2^ = 0.474, Durbin-Watson = 1.721					
IL-6	Age	155.992	0.233	2.893	0.005	26.153 *
	PM	394.223	0.588	7.310	<0.001	
	Exercise	−220.052	−0.328	−4.081	<0.001	
	*R* = 0.713, *R*^2^ = 0.508, Adjusted *R*^2^ = 0.489, Durbin-Watson = 2.039					
TNF-α	Age	66.327	0.095	1.114	0.269	20.636 *
	PM	418.062	0.598	7.022	<0.001	
	Exercise	−200.568	−0.287	−3.369	0.001	
	*R* = 0.670, *R*^2^ = 0.449, Adjusted *R*^2^ = 0.427, Durbin-Watson = 1.611					

8-OHdG: 8-hydroxydeoxyguanosine, MDA: malondialdehyde, SOD: superoxide dismutase, CAT: catalase, IL-1β: interleukin-1β, IL-6: interleukin-6, TNF-α: tumor necrosis factor α, * *p* < 0.05.

## Data Availability

Data generated and analyzed during this study are included in this article. Additional data are available from the corresponding author on request.
